# Characterizing geometric distortions of 3D sequences in clinical head MRI

**DOI:** 10.1007/s10334-022-01020-8

**Published:** 2022-06-03

**Authors:** Katri Nousiainen, Teemu Mäkelä, Juha I. Peltonen

**Affiliations:** 1 HUS Medical Imaging Center, Helsinki University Hospital and University of Helsinki, Helsinki, Finland; 2 Department of Physics, University of Helsinki, Helsinki, Finland

**Keywords:** Magnetic resonance imaging, Artifacts, Quality control, Healthcare quality assurance

## Abstract

**Objective:**

Phantoms are often used to estimate the geometric accuracy in magnetic resonance imaging (MRI). However, the distortions may differ between anatomical and phantom images. This study aimed to investigate the applicability of a phantom-based and a test-subject-based method in evaluating geometric distortion present in clinical head-imaging sequences.

**Materials and methods:**

We imaged a 3D-printed phantom and test subjects with two MRI scanners using two clinical head-imaging 3D sequences with varying patient-table positions and receiver bandwidths. The geometric distortions were evaluated through nonrigid registrations: the displaced acquisitions were compared against the ideal isocenter positioning, and the varied bandwidth volumes against the volume with the highest bandwidth. The phantom acquisitions were also registered to a computed tomography scan.

**Results:**

Geometric distortion magnitudes increased with larger table displacements and were in good agreement between the phantom and test-subject acquisitions. The effect of increased distortions with decreasing receiver bandwidth was more prominent for test-subject acquisitions.

**Conclusion:**

Presented results emphasize the sensitivity of the geometric accuracy to positioning and imaging parameters. Phantom limitations may become an issue with some sequence types, encouraging the use of anatomical images for evaluating the geometric accuracy.

## Introduction

Image artefacts are an inseparable part of magnetic resonance imaging (MRI). The geometric distortions in MRI are often subtle and, hence, ignored in many diagnostic tasks. Simultaneously, several image-guided medical operations, such as MRI-only radiotherapy planning (RTP), stereotactic radiosurgery (SRS), or stereotactic neurosurgery, require geometrically accurate images to enable precise treatment. Unexpected changes in geometric accuracy may also indicate scanner malfunction. Thus, the severity of the geometric distortions present in the clinical MRI should be evaluated, minimized, and monitored.

The geometric distortions in MRI arise from both system-related and patient-specific sources. The system-related distortions consist of the main magnetic field (B_0_-field) inhomogeneity, gradient nonlinearity (GNL), and eddy currents, whereas patient-specific distortions include susceptibility and water-fat shift (WFS) effects. The distortions become relevant when the image geometry forms a basis for a medical operation. For example, the distortions have a potential to corrupt SRS [1] and MRI-only RTP of tangential whole breast intensity-modulated radiotherapy [2]. The geometric distortions no longer prevent MRI-only RTP in the pelvic region, as system-related distortions can be measured and partly corrected, however the distortion magnitudes should always be evaluated [3]. According to American Association of Physicists in Medicine (AAPM) 2021 report [4], in MRI-simulation of external beam radiotherapy, the total system-related distortions should be less than 2 mm in 25 cm diameter of spherical volume. For SRS, a maximum distortion of 1 mm or 1.5 mm in planning target volume of < 2 cm or > 2 cm in diameter, respectively, have been reported appropriate [5]. In addition, Institute of Physics and Engineering in Medicine (IPEM) topical report 2021 [6] states that reducing radiotherapy-related side-effects can be equally as important as the survival outcomes of the cancer treatments. The distortions should also be controlled when they could potentially contribute inaccuracies to MRI-CT-co-registration, for instance in neuronavigation, MRI-only surgical planning [7], fusion between pre- and intraoperative MRI [8], or robot-assisted surgery already reaching sub-millimeter movement precision [9]. Minimizing the geometric distortions reduces the total error of the abovementioned treatments and should result in better treatment outcomes.

A typical MRI quality assurance (QA) protocol includes at least a rudimentary evaluation of the geometric distortions, typically by imaging a phantom of known dimension. For example, in American College of Radiology’s (ACR) accreditation program a deviation from the nominal phantom diameter is measured [10]. Guidelines for MRI scanners used in RTP encourage the distortions to be measured over a larger field-of-view (FOV) [4, 11]. The distortions can be measured by comparing the MRI acquisition to a geometrically accurate reference, for example a virtual reference phantom or a computed tomography (CT) scan of the phantom [4]. A displacement field and distortion magnitudes are often calculated through control-point detection [e.g., 12–14], or via nonrigid registration [e.g., 15, 16]. The accuracy requirements of RTP require excellent B_0_-field homogeneity [4, 6, 17], which can be evaluated by B_0_-field mapping [18–20]. B_0_-field inhomogeneities and GNL are often assumed to be smallest near the scanner isocenter, patient-specific distortions being dominant in this region; thus, the B_0_-field mapping can be used to obtain patient susceptibility-induced distortions near scanner isocenter [e.g., 21–23]. A method also exists for differentiating between the B_0_-field inhomogeneities and GNL effect in the frequency encoding direction [15, 19, 24]. The methods for characterizing different distortion sources can be used for instance for patient-specific unwarping of MRI volumes [e.g., 19].

The magnitudes of GNL-related geometric distortions are proportional to gradient field strength and, consequently, inversely proportional to the receiver bandwidth (rBW). Higher rBW reduces signal-to-noise ratio (SNR), and thus, many MRI-only RTP commissioning and feasibility studies involve rBW optimization [e.g., 25]. Furthermore, the dosimetric effect of different rBWs have been studied with phantom measurements and simulations [26–28]. According to AAPM report 2021 [4] rBW must be greater than 220 Hz per pixel at 1.5 T and 440 Hz per pixel at 3 T in RTP, so that WFS is less than one pixel, and according to IPEM topical report 2021 [6], WFS should be reduced to displacements of 1 mm or less for RTP. However, even an optimized rBW does not remove the effect from GNL and eddy-current-induced magnetic fields. The GNL-related distortions can be moderated by applying a correction algorithm suppressing the distortions [29–32], and thus, most scanner vendors nowadays offer intrinsic geometric distortion correction.

In MRI-simulation, the center of the volume-of-interest (VOI) should be positioned at the scanner isocenter [4, 6]. The isocenter position could vary relative to a specific anatomic site because of inter-operator variance in patient positioning, coil design, differing imaging indications, FOV-positioning mode, or technical issues such as table movement imprecision. Geometric fidelity and length of FOV can be improved by stitching of ideally centered acquisitions [4], and an acquisition with a moving patient table can extend the imaging volume in table-movement-direction and reduce geometric distortions compared to static imaging [15]. The fidelity of a phantom QA workflow for RTP has been tested by purposefully misplacing a large-FOV phantom relative to the isocenter and deemed robust for small misplacements [16]. The B_0_-field homogeneity, and consequently geometric accuracy, can be improved trough shimming; however, local shimming can reduce the geometric accuracy elsewhere [28].

In an earlier work, our group has presented MRI scanner QA methods based on diagnostic 3D FLAIR (FLuid-Attenuated Inversion Recovery) acquisitions [33], yet the geometric fidelity of the images was not assessed. In addition, we have previously studied the geometric accuracy of MPRAGE (Magnetization Prepared—RApid Gradient Echo) acquisitions on 12 MRI scanners with a 3D-printed phantom and a nonrigid-registration-based analysis [34], but no test-subject acquisitions were utilized. The geometric distortion in MRI have been widely studied, however, the geometric distortions are rarely evaluated in anatomical images for QA purposes despite their potential additional distortion sources compared with phantom studies.

The aims of this study were: (1) to characterize the changes in the geometric accuracy of two common head-imaging sequences, 3D FLAIR and MPRAGE, when the rBW and the patient-table position were varied, (2) to investigate the applicability of non-rigid registration and a 3D-printed phantom for this purpose, and (3) to determine if test-subject-based distortion evaluation add benefit to the phantom investigations.

## Materials and methods

### Image acquisition

We imaged a 3D-printed grid phantom and test subjects on a 1.5 T MRI scanner (MAGNETOM Aera, Siemens Healthineers, Erlangen, Germany) and on a 3 T MRI scanner (MAGNETOM Skyra, Siemens Healthineers, Erlangen, Germany) using two 3D sequences with varying rBW and patient-table positions. The sequences were sagittal T1-weighted MPRAGE and 3D FLAIR, which is based on 3D SPACE (Sampling Perfection with Application optimized Contrasts by using different flip angle Evolutions) [35]. Table 1 provides imaging parameters for the baseline sequences that are in routine clinical use. The volumes with varied table positions were reconstructed without the scanners’ user-selectable geometric distortion correction, whereas the volumes of varying rBW were reconstructed both with and without the scanners’ 3D distortion correction (DIS3D).

**Table 1:** The imaging parameters. The presented receiver bandwidth is used in the routine clinical setting. In addition, volumes with receiver bandwidth of 250, 300, 500, 750, and 815 Hz/px were acquired. Here, *SI* superior-inferior, voxel sizes are given as phase encoding (PE) × frequency encoding (FE) × slice encoding direction, and the acquisition matrix and the field of view in PE × FE direction

Scanner field strength (*T*)	1.5		3	
Sequence name	MPRAGE	3D FLAIR	MPRAGE	3D FLAIR
Repetition time (ms)	2200	5000	2300	5000
Echo time (ms)	1.13	335	2.32	388
Inversion time (ms)	900	1600	900	1600
Echo train length	144	242	224	278
Parallel imaging factor	2	2	2	2
Flip angle (°)	8	120	8	120
Receiver bandwidth (Hz/px)	150	592	200	750
Frequency encoding direction	SI	SI	SI	SI
Acquisition voxel size (mm^3^)	1.0 × 1.0 × 1.2	1.1 × 1.0 × 1.3	1.0 × 1.0 × 0.9	1.0 × 1.0 × 1.5
Reconstruction voxel size (mm^3^)	1.0 × 1.0 × 1.0	1.0 × 1.0 × 1.0	1.0 × 1.0 × 0.9	0.5 × 0.5 × 1.2
Acquisition matrix	246 × 256	216 × 256	256 × 256	256 × 256
Field of view (mm × mm)	250 × 250	234 × 250	250 × 250	250 × 250
Partial Fourier	No	PE: Auto^a^ FE: No	No	PE: Auto^a^ FE: 7/8
Spectral fat saturation	No	Yes	No	Yes

^a^The partial Fourier factor in the PE direction was chosen automatically by the scanner for the 3D FLAIR sequences and not obtainable for the user

The phantom grid was 3D printed from polylactide filament, and it was formed by 3-mm-thick solid bars that covered a volume of 12 cm in height and 15 cm × 15 cm in width. The grid was placed in a drum (17 cm in height and 20 cm in diameter) that was filled with mineral oil. Further details of the phantom are presented in [34].

With the phantom, the scanner isocenter was set in the middle of the phantom grid, and with the test subjects, in the middle of the corpus callosum. We set the imaging orientation to non-oblique sagittal, so that the imaging volume was orthogonal relative to the main magnetic field direction. We acquired a baseline volume with the routinely used rBW (see Table 1) followed by acquisitions with varying rBW of values 250, 300, 500, 750, and 815 Hz/px, hereafter referred as rBW sweep. In addition, we acquired seven acquisitions with the routinely used rBW but at different patient-table positions, hereafter referred as offset sweep. The table positions for the offset sweep were − 60 mm, − 40 mm, − 20 mm, 0 mm, + 20 mm, + 40 mm, and + 60 mm from the original position, so that negative values moved the isocenter toward the feet (F) and positive values towards the head (H). The anatomical FOV and imaging origin were constant during the offset sweep, which was achieved by first moving the patient table, and then setting the imaging origin back to the original anatomical location. No table movement was allowed after this. Altogether, these acquisitions resulted in the following datasets for both scanners, where a single test subject (two individuals) was used per each rBW or offset sweep set:

1. MPRAGE, phantom, rBW sweep
2. MPRAGE, phantom, offset sweep
3. 3D FLAIR, phantom, rBW sweep
4. 3D FLAIR, phantom, offset sweep
5. MPRAGE, test subject, rBW sweep
6. MPRAGE, test subject, offset sweep
7. 3D FLAIR, test subject, rBW sweep
8. 3D FLAIR, test subject, offset sweep

The co-operative test subjects were instructed to stay as still as possible during the acquisitions. An ethical approval for the test subject acquisitions was granted by the Ethics Committee of Hospital District of Helsinki and Uusimaa.

In addition, the phantom was scanned with CT (SOMATOM Definition Edge, Siemens Healthineers, Erlangen, Germany) with 120 kV tube voltage, 191 mA tube current, and 0.5 mm slice thickness, and reconstructed with 0.4 mm × 0.4 mm pixel size, 512 × 512 image matrix, and general-purpose soft-tissue J45s-kernel. This CT scan was the same as used in [34], but we performed a QA CT scan with similar settings before the MRI acquisitions to affirm that the phantom had remained unchanged.

### Image analysis

**Fig. 1:**
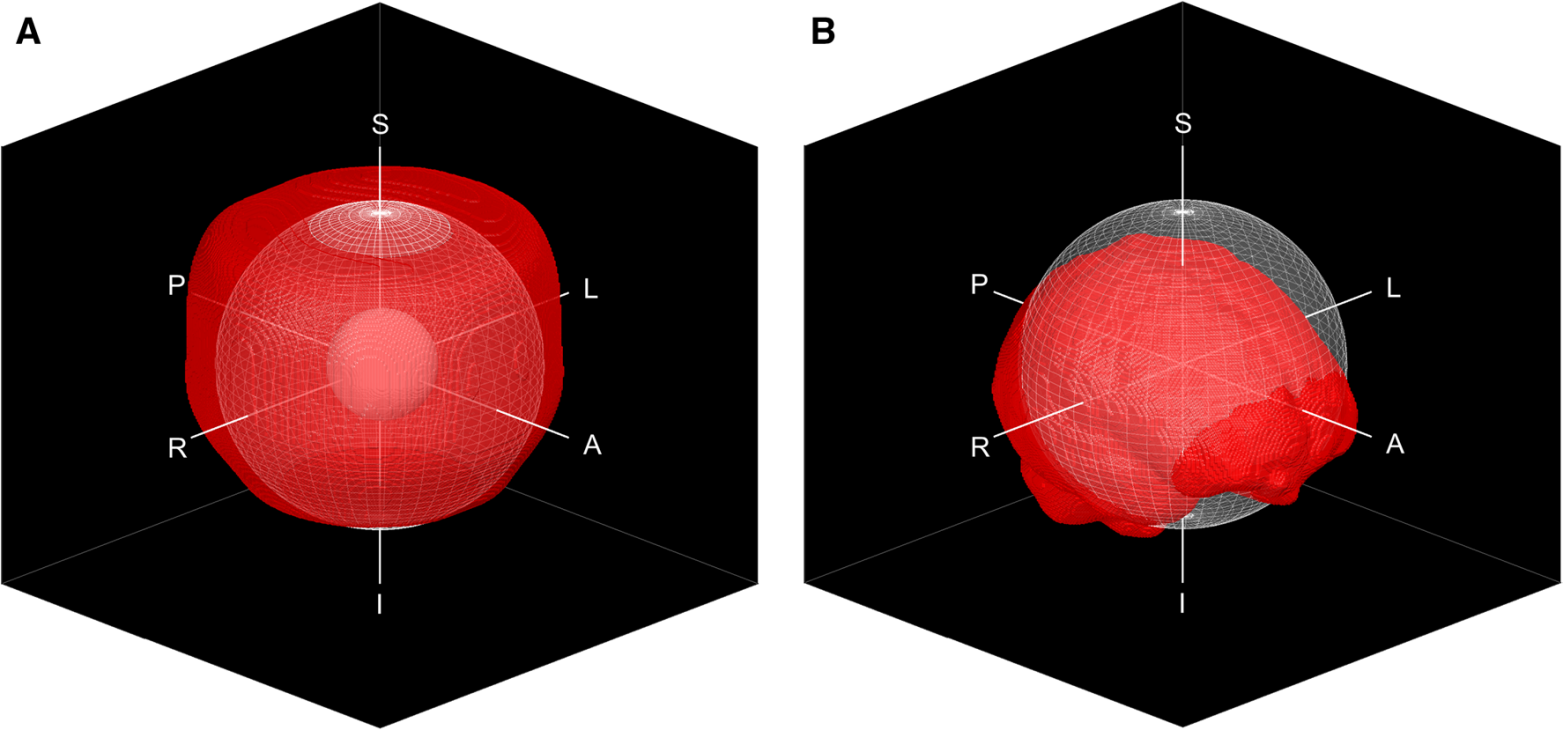
**a** The signal producing area of the phantom is shown in red with a solid and a wired sphere, which represent the 27-mm-radius volume-of-interest (VOI) used in the rigid registrations, and the 80-mm-radius VOI, respectively. **b** A brain mask (red) with the superimposed 80-mm-radius VOI (white). Here, *R* right, *L* left, *A* anterior, *P* posterior, *S* superior, *I* inferior

The image analysis utilized 3D Slicer image-processing platform version 4.8 [36], with BRAINSFit module [37], and a nonrigid-registration toolbox elastix [38, 39]. All the MRI volumes were rigidly registered to a corresponding reference acquisition: offset-sweep volumes to the centered volume (0 mm offset) and the rBW-sweep volumes to the volume with the highest rBW (815 Hz/px). In the rigid registrations, we used a 27-mm-radius spherical VOI around the imaging origin for the phantom volumes (see Fig. 1a), and brain masks with the test subject acquisitions (Fig. 1b). Next, the MRI volumes were registered to the corresponding reference acquisition with a deformable registration using elastix with the toolbox’s default B-Spline parameter file, where the final grid spacing was set to 30 mm. For the nonrigid registration, we used masks that covered the signal producing area of the phantom (Fig. 1a) or the test subject’s head. This procedure resulted in displaced to centered and varied-*rBW* to high-*rBW* MRI-to-MRI registrations for both the phantom and the test-subject acquisitions. With rBW-sweep volumes, the registration workflow was performed for both the DIS3D and the uncorrected volumes, so that the reference volume for the DIS3D volumes was also a corrected one and for the uncorrected volumes an uncorrected one.

The phantom MRI volumes were also registered with the CT scan. The CT volume was rigidly registered to the MRI volume in two steps: first time roughly by the center-of-masses, and second time with the 27-mm-radius spherical VOI. After the two rigid registrations, the MRI volume was nonrigidly registered to the CT volume, resulting in MRI-to-CT registrations for the phantom. The MRI-to-CT phantom registrations represented the underlying absolute distortions present in the MRI acquisitions with the CT volume acting as a ground truth, whereas the MRI-to-MRI registration resulted in relative distortions.

The nonrigid registrations produced displacement vector fields—effectively interpolated between the grid lines in the phantom acquisitions—that we utilized in the further analysis using MATLAB version 2020b (The MathWorks, Natick, Massachusetts, USA). By convention, the registration steps resulted in zero displacement at the imaging origin: the displacement fields were normalized to the imaging origin by subtracting the origin’s displacement vector from the displacement field (effectively a third rigid registration without rotation). All further analyses were based on the distortion magnitudes, that is, the Euclidean norm of the displacement field. Next, we recorded the distortions as a function of the distance from the imaging origin, and calculated the mean, median, and 25th, 75th and 95th percentile values in one-mm-thick sphere surfaces at different radii. In addition, we made a linear fit to the radial distortion data (limited within an 80-mm-radius sphere) and recorded the slope of the linear fit indicating an average rate-of-change in the distortions. The linear fit was performed with a linear regression model without the intercept term; thus, the fit was forced through the origin.

We also evaluated inter-acquisition movement by calculating the mean magnitude of the translation inside an 80-mm-radius VOI for the translations that resulted from the rigid MRI-to-MRI registrations.

## Results

**Fig. 2:**
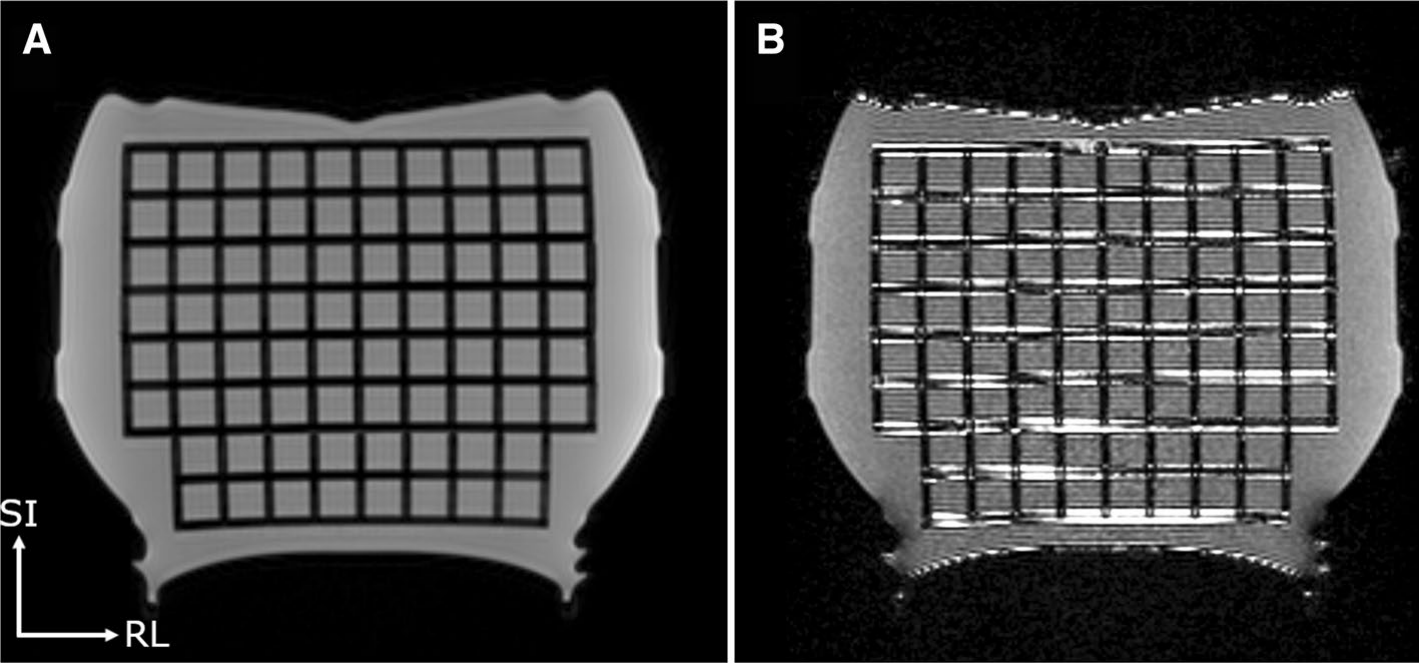
A coronal-slice image of the phantom with **a** MPRAGE (receiver bandwidth 150 Hz/px) and **b** 3D FLAIR (250 Hz/px) sequence on the 1.5 T scanner without the 3D distortion correction. The phantom FLAIR images suffered from high-intensity artefacts. Here, *SI* superior–inferior (i.e., frequency encoding) direction and *RL* right–left (i.e., slice encoding) direction

Figure 2 shows coronal images of the phantom on the 1.5 T scanner with uncorrected MPRAGE (rBW = 150 Hz/px) and 3D FLAIR (rBW = 250 Hz/px) sequences. The latter suffers from high-intensity variations at the grid edges, which was the case for all the 3D FLAIR phantom acquisitions.

**Fig. 3:**
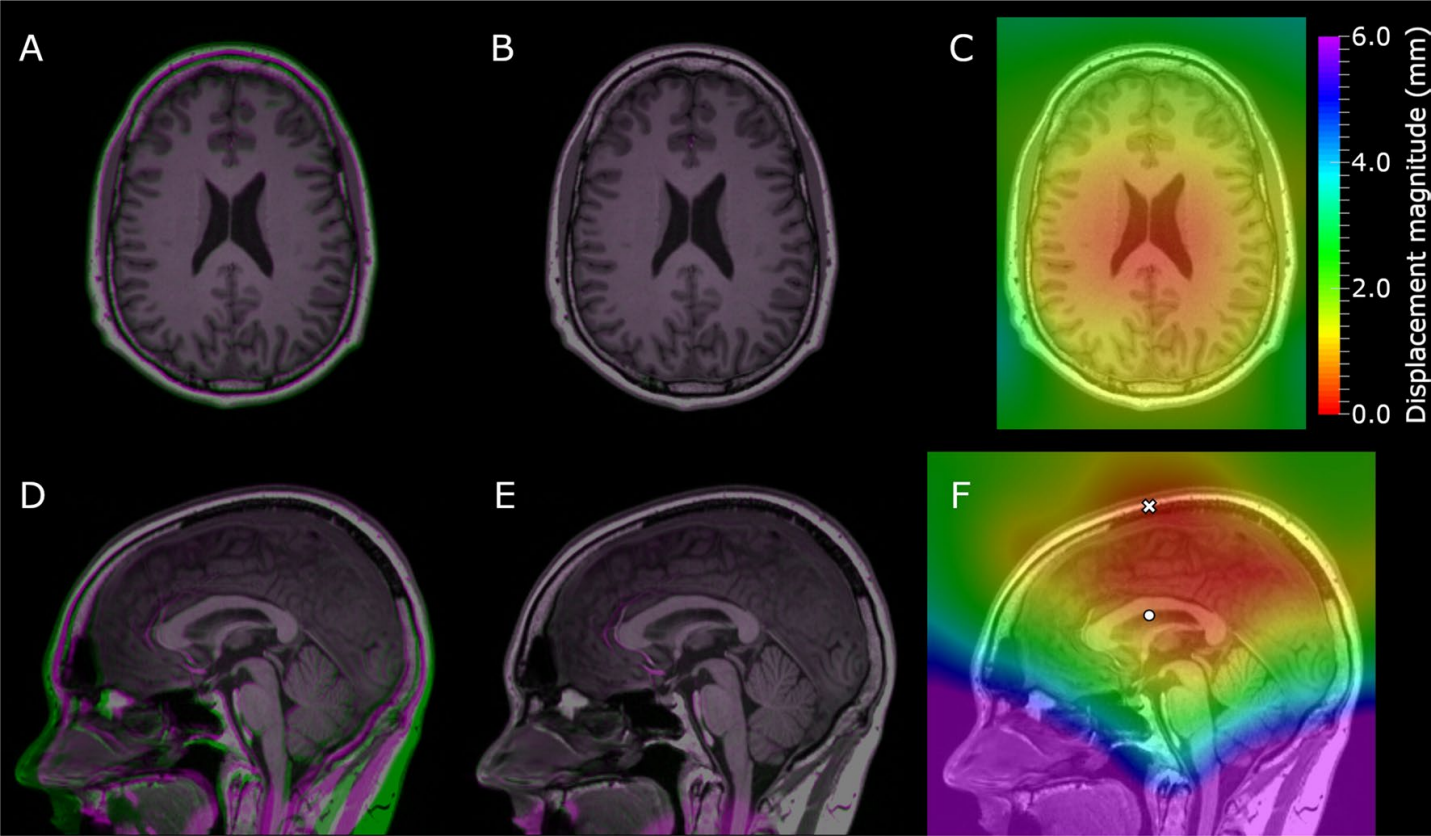
The axial (**a**–**c**) and sagittal (**d**–**f**) slices of the offset-sweep test-subject acquisitions with MPRAGE sequence on the 3 T scanner. Here, **a** and **d** show the centered (0 cm offset) volume in purple and the H60 cm offset volume in green after the rigid and before the elastic registration. Similarly, **b** and **e** show the volumes after the elastic registration. **c** and **f** show the resulting displacement magnitude map on the centered volume. In **f**, the circle denotes isocenter location at the 0 cm offset and the cross at the H60 mm offset

Figure 3 shows examples of axial and sagittal slices for the H60 cm offset test-subject volume (MPRAGE sequence on the 3 T scanner) before and after the nonrigid registration to the centered volume together with a resulting displacement magnitude map.

**Fig. 4:**
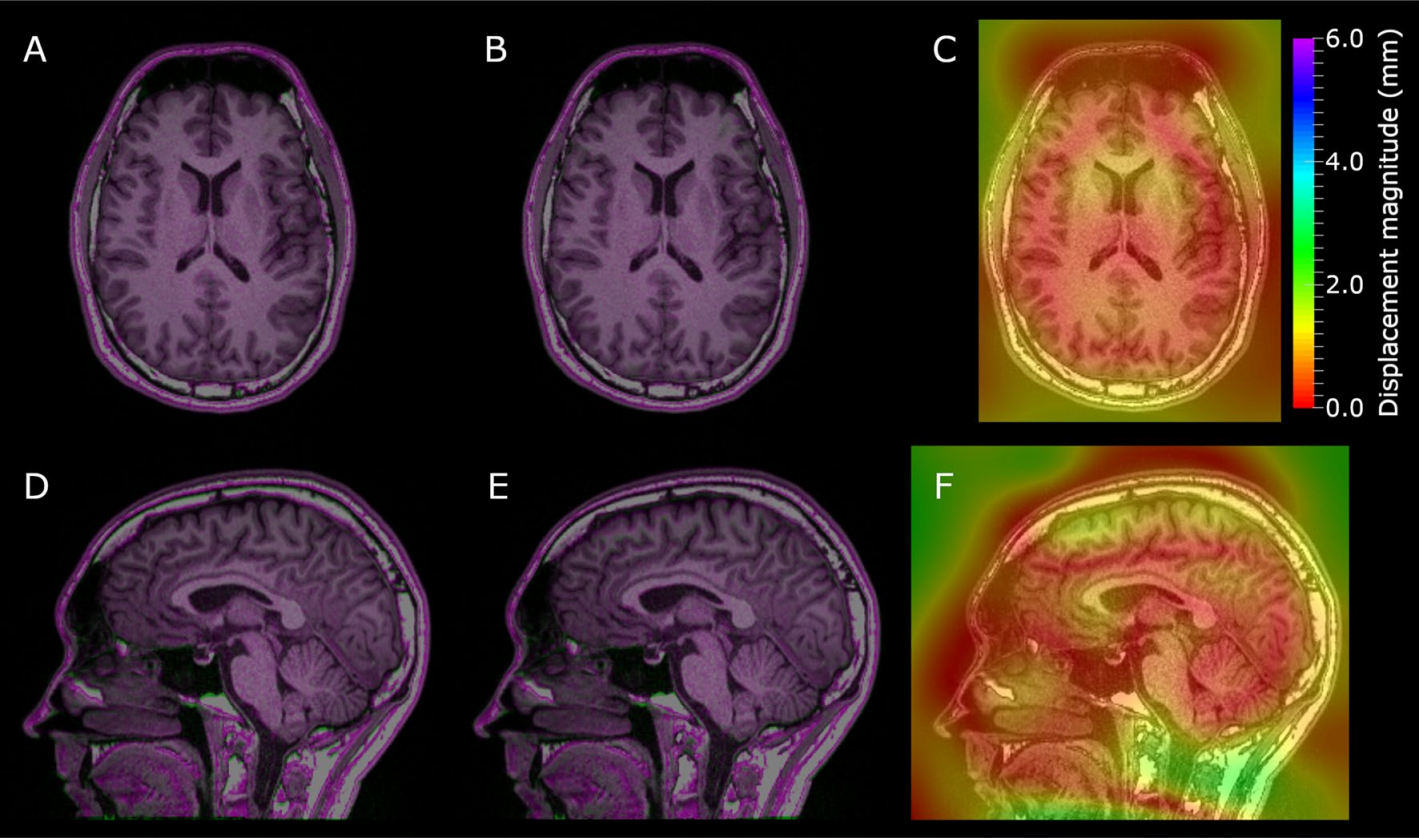
The axial (**a**–**c**) and sagittal (**d**–**f**) slices of the rBW-sweep test-subject acquisitions with uncorrected MPRAGE sequence. Here, **a** and **d** show the high-rBW (815 Hz/px) volume in purple and the 200 Hz/px volume in green after the rigid and before the elastic registration. Similarly, **b** and **e** show the volumes after the elastic registration. **c** and **f** show the resulting displacement magnitude map on the high-rBW volume

Figure 4 shows examples of the axial and sagittal slices for the 200 Hz/px rBW test-subject volume (uncorrected MPRAGE sequence on the 3 T scanner) before and after the elastic registration to the 815 Hz/px volume together with a resulting displacement magnitude map.

For the rBW sweep, the mean magnitude of the translation from the rigid MRI-to-MRI registrations was between 0.0 and 0.3 mm for the phantom acquisitions and between 0.3 and 1.9 mm for the test-subject acquisitions. For the offset sweep, the mean translations magnitudes were 0.1–1.1 mm and 0.4–3.2 mm for the phantom acquisitions and the test-subject acquisitions, respectively.

**Fig. 5:**
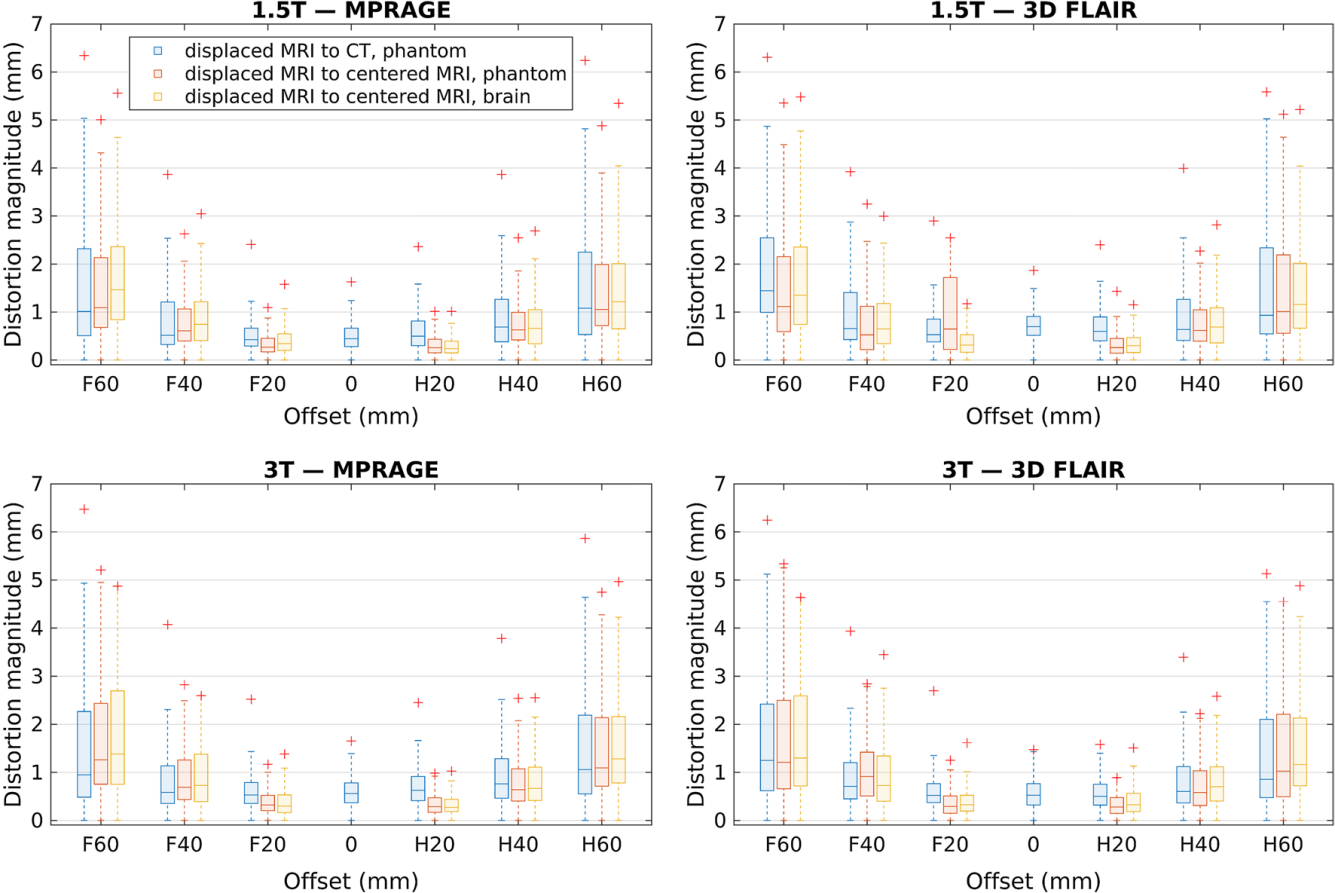
Distortion distributions in the 80-mm-radius spherical VOI for offset-sweep acquisitions. Here the line is the median value, the box defines the range from 25 to 75th percentile, and the “+” marks the maximum value

Figure 5 shows boxplots of the distortion magnitude distributions produced by offset-sweep registrations (displaced phantom-MRI to phantom-CT, displaced phantom-MRI to centered phantom-MRI, and displaced brain-MRI to centered brain-MRI) for both scanners and sequences. The overall distortion magnitudes increase with larger offsets, and the distribution broadens simultaneously. For the MRI-to-CT phantom registrations, 0, F20, and H20 mm offsets show the same level of distortions. With higher absolute offsets, the distortion distributions are similar between different registrations within the offset in question. Additionally, the distributions are nearly pairwise symmetrical around 0 mm offset.

**Fig. 6:**
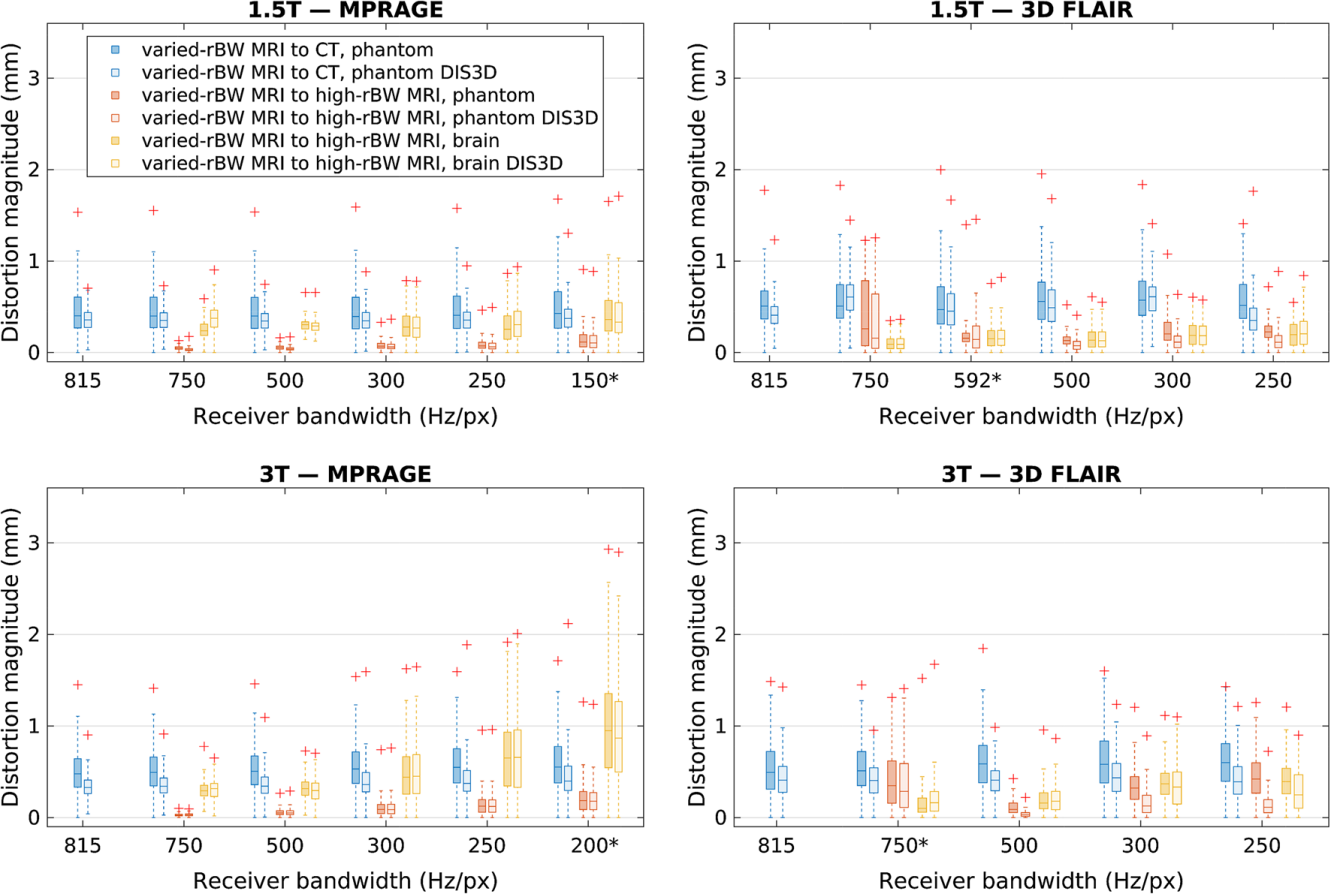
Distortion distributions in the 80-mm-radius spherical VOI for rBW-sweep acquisitions. Here, the line is the median value, the box defines the range from 25 to 75th percentile, and the “+” marks the maximum value. DIS3D denotes 3D distortions corrected acquisitions. *rBW* receiver bandwidth, high rBW = 815 Hz/px, and the asterisks (*) denotes the routinely used rBW. ﻿The acquisition voxel size in the frequency encoding direction (i.e., the direction of the readout gradient) was 1.0 mm with all the acquisitions

Figure 6 shows boxplots of the distortion magnitude distributions produced by rBW-sweep registrations (varied-rBW phantom-MRI to phantom-CT, varied-rBW phantom-MRI to high-rBW phantom-MRI, and varied-rBW brain-MRI to high-rBW brain-MRI) for both scanners and sequences. Both the DIS3D and the uncorrected volumes are presented. With varied-rBW to high-rBW MRI-to-MRI registrations, the overall distortion magnitudes increase with decreasing rBW, and the distributions broaden for both brain and phantom acquisitions, except for the 3D FLAIR acquisitions with rBW = 750 Hz/px on both scanners. At least part of the discrepancy in the 3D FLAIR results can be attributed to the registration being affected by the high-intensity artefacts (Fig. 2b). For the MRI-to-CT phantom registrations, the distortion magnitude distributions remain generally constant regardless of the rBW primarily due to the lack of the WFS effect in the phantom. The overall distortion magnitude is smallest for the varied-rBW phantom-MRI to high-rBW phantom-MRI registrations. In general, the distortion correction reduced the median and maximum distortions for the MRI-to-CT phantom registrations that represent the absolute distortions. For the MRI-to-MRI registrations that represent the relative distortions, the effect of the correction was present in the 3D FLAIR phantom registrations, but not in the test-subject acquisitions with exception of the rBW 250 Hz/px on the 3 T scanner. The distortion correction did not have much effect on any of the MPRAGE MRI-to-MRI registrations.

**Fig. 7:**
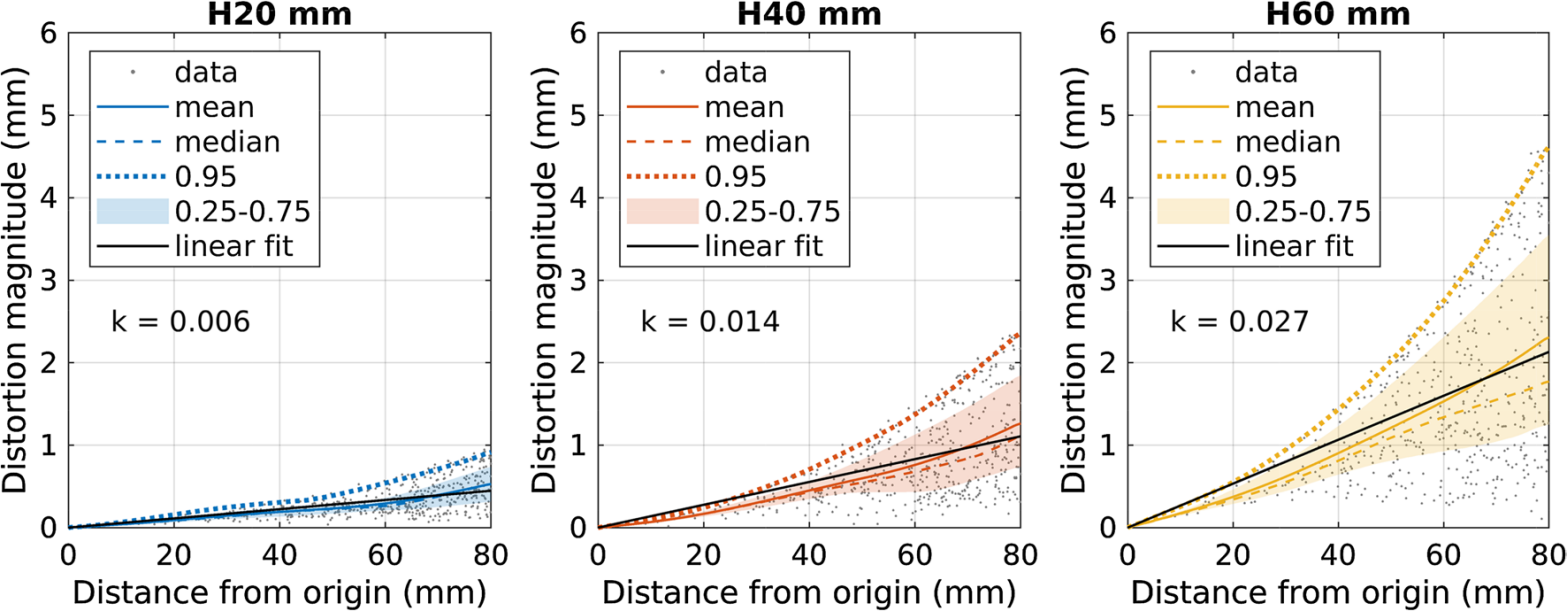
Examples of mean and median distortion magnitudes versus distance from the imaging origin (the same anatomical location in each graph) for the offset sweep. Here, H20 mm, H40 mm, and H60 mm offsets for displaced brain-MRI to centered brain-MRI registrations of MPRAGE acquisitions on the 3 T scanner are shown. In addition, every 5000th data point, 95th percentile (0.95), 25th–75th percentile interval (0.25–0.75), and linear regression fit to the radial data in 0–80 mm distance interval with corresponding slope (*k*) are visible

Figure 7 shows examples of the distortion magnitudes as a function of the distance from the imaging origin for three different table offsets in the MRI-to-MRI brain registrations. The anatomical location of the imaging origin is the same for each case. The distortion maximum as well as the slope of the linear fit increases with larger offset.

**Fig. 8:**
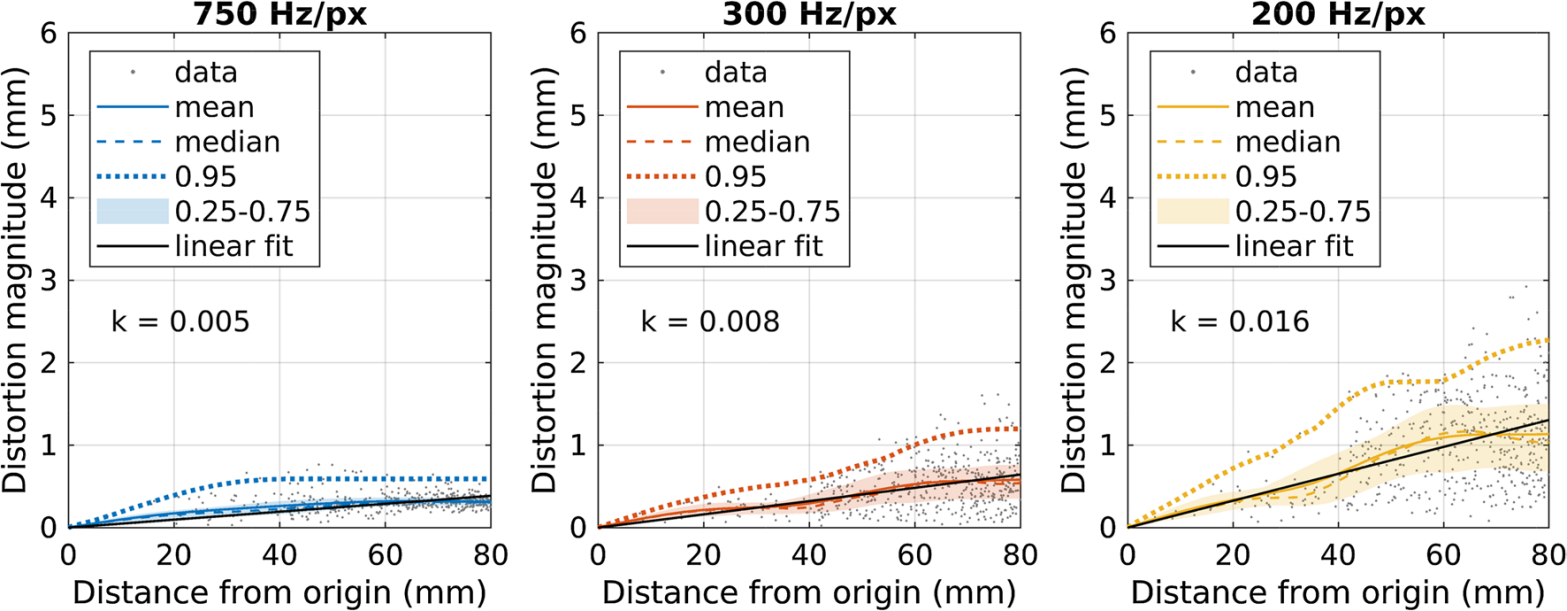
Examples of mean and median distortion magnitudes versus distance from the imaging origin (the same anatomical location in each graph) for the rBW sweep. Here, 750 Hz/px, 300 Hz/px, and 200 Hz/px mm rBW for varied-rBW brain-MRI to high-rBW brain-MRI registrations of uncorrected MPRAGE acquisitions on the 3 T scanner are shown. In addition, every 5000th data point, 95th percentile (0.95), 25th–75th percentile interval (0.25–0.75), and linear regression fit to the radial data in 0–80-mm distance interval with corresponding slope (*k*) are visible. ﻿The acquisition voxel size in the frequency encoding direction (i.e., the direction of the readout gradient) was 1.0 mm with all the acquisitions

Figure 8 shows examples of the distortion magnitudes as a function of the distance from the imaging origin for three different rBW settings in the MRI-to-MRI brain registrations. The anatomical location of the imaging origin is the same for each case. The distortion maximum as well as the slope of the linear fit increases with smaller rBW.

**Fig. 9:**
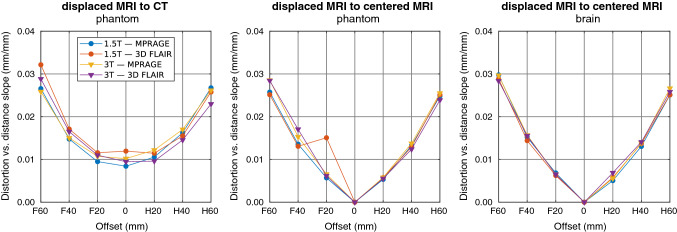
The slopes of the linear fits to radial distortion data (in the 80-mm-radius volume-of-interest) versus the offset-sweep volume for different registrations of the volumes. The hypothetical 0-mm-offset data point is added to the MRI-to-MRI graphs for illustrational purposes

Figure 9 summarizes the slopes of the linear fits to the radial distortion data from offset-sweep registrations. The slopes are nearly symmetrical around the 0 mm offset. Some irregularities can be seen in the 3D FLAIR volumes, attributable to the registration being affected by the high-intensity artefacts. The slope values are similar for all the registrations regardless of the scanner or the sequence, and the phantom and test-subject acquisitions agree well with each other. The increasing slopes show that not only the magnitudes, but also the rate-of-change in distortions grow when imaging target is moved away from the isocenter (i.e., 0 mm offset).

**Fig. 10:**
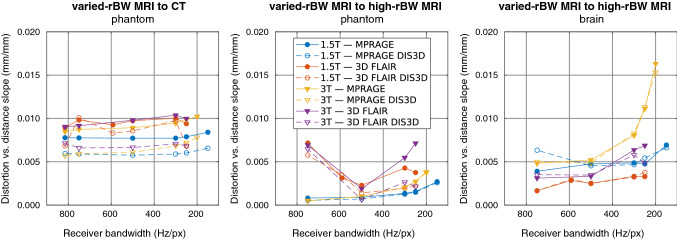
The slopes of the linear fits to radial distortion data (in the 80-mm-radius volume-of-interest) versus the rBW-sweep volume for different registrations of the volumes. DIS3D denotes 3D distortions corrected acquisitions. *rBW* receiver bandwidth, and high rBW = 815 Hz/px

Figure 10 shows the slopes of the linear fits to radial distortion data from rBW-sweep registrations for both the DIS3D and the uncorrected volumes. The slopes from MPRAGE acquisitions and 3D FLAIR acquisitions with the test subject mainly increase with decreasing rBW, yet the relation is nonlinear. The slopes from 3D FLAIR acquisitions and MRI-to-CT phantom registrations do not follow a clear trend. The slopes from 3D FLAIR acquisition with varied-rBW phantom-MRI to high-rBW phantom-MRI registrations have a higher slope value with rBW = 750 Hz/px than with the other rBW values. The slopes decrease with the distortion correction in the MRI-to-CT registrations but are similar between the DIS3D and the uncorrected volumes in the MRI-to-MRI registrations.

## Discussion

We investigated geometric distortions in clinical head-imaging and studied if phantom and test-subject based distortion measurements yield comparable results. We characterized two 3D sequences typically used in head MRI on a 1.5 T and a 3 T scanner by varying the patient-table position and the rBW settings. The phantom volumes registered to a CT volume gives an estimate of the underlying absolute accuracy of the acquisitions, and the elastic co-registration between MRI volumes provide an estimate for the relative increase in distortions due to varying imaging settings. In general, the 3D-printed phantom showed comparable results to the test-subject acquisitions but suffered from artefacts in the 3D FLAIR images and could not detect changes in all situations (e.g., the 3 T MPRAGE at low rBW primarily due to WFS). These limitations should be considered when utilizing phantoms in the evaluation of the geometric accuracy for clinical purposes. The non-rigid registration method could be used for example in the rBW optimization if the impact on the geometric accuracy needs to be evaluated.

By changing the patient-table position, we obtained volumes with varying distortions and achieved a very good correspondence between the phantom and the test-subject acquisitions. Only minor changes on the absolute (phantom MRI-to-CT) and relative (MRI-to-MRI) distortions were detected within ± 20 mm offsets indicating that — with the studied scanners — mispositioning from the isocenter would not cause major issues at this scale. When the imaging origin was located ± 40 and ± 60 mm from the isocenter, the geometric distortions increased progressively. With ± 60 mm difference between the imaging origin and the isocenter, even the median distortion exceeded the acquisition pixel size, with maximum values of 5 mm and more. It should be noted that we reported displacement field magnitudes: in the worst case the error in a distance measurement (e.g., phantom diameter used in many QA guidelines) could be up to twice the reported value at a specific radius, if the distortions were in the opposite directions. We observed small differences in the maximum values between the absolute and relative distortions, whereas the relative phantom and test-subject results were in good agreement. These results imply that the effect of the isocenter mispositioning on the distortions can be evaluated with both the phantom and the test-subject measurements.

Based on the findings, the positioning of the patient is a key contributor in geometric-distortion minimization. The possibility that the images could later be used in surgical planning, or other high precision applications, should also be taken into specific consideration in the QA protocol. To avoid misalignment of the isocenter and the imaging origin, imaging sites should have clear study instructions for ensuring both the selection of the correct sequences and the appropriate image positioning. An automatic alignment of the imaging volume and an isocenter verification after initial scout images are potential ways to increase scan repeatability. The phenomenon we observed in the offset sweep also emphasizes the necessity for QA of the positioning lasers and table-movement accuracy in RTP and other location sensitive applications. A continuous monitoring of the distortions could provide warnings for scanner malfunctions as well as aid in avoiding mishaps after protocol optimization and system updates or maintenance.

By varying the rBW, we managed to identify a situation with the MPRAGE sequence, where both absolute and relative phantom results diverged notably from the test-subject acquisitions. As can be seen in Fig. 6, especially with 3 T, the phantom distortions remain fairly constant while test-subject acquisitions show strong rBW correlation. The maximum distortion values of these clinical acquisitions correspond well with theoretical WFS (e.g., 1.5 mm at 1.5 T with 150 Hz/px, and 2.2 mm at 3 T with 200 Hz/px), suggesting that WFS is the main error contributor. The 3D FLAIR acquisitions with built-in fat-saturation are also less affected by the susceptibility or field inhomogeneity induced distortion than the MPRAGE acquisitions. Likewise, our phantom measurements were limited to the submerged grid and therefore susceptibility effects, also dependent on the rBW, from the air boundaries were minimal. The usage of a simple phantom could lead to the underestimation of these distortions compared to the presented test-subject-based method.

The scanner manufacturers offer geometric distortion corrections that are recommended to be enabled with relevant imaging protocols. The effect of the vendor-provided distortion correction was studied by comparing the corrected and uncorrected volumes with variable rBW. In the MRI-to-CT phantom registrations, the distortions were substantially lower after the correction was applied. A slightly more prominent effect was observed for 3D FLAIR than MPRAGE on the test-subject registrations, especially with the 3 T scanner. With the test subjects, the effect of other error sources in addition to GNL likely increases limiting the effect of the achieved distortion correction. Thus, only the 3D FLAIR test-subject acquisition with 250 Hz/px rBW (with the strongest gradient fields) was notably affected by the correction. Overall, the effect was minimal for the MRI-to-MRI registrations, as could be expected, because the uncorrected volumes were registered to uncorrected references and the corrected volumes to the corrected references. This consistency acts as an internal validation: results did not depend on small changes in the initial conditions. We did not investigate the effect of the vendor-provided distortion correction on the offset-sweep volumes, because the correction could be applied only to a limited volume around the scanner isocenter.

While the overall distortions magnitudes were generally inversely proportional to the rBW, non-linear response was also observed. The non-linearity may reflect the internal response of the imaging sequence to imaging parameter changes: sequences include various optimizations to guarantee image quality with different imaging settings, thus, it is likely that the change of rBW will also affect other parameters. For example, SNR is often a major driving factor of the sequence behavior, which may lead to unexpected results in terms of distortion [35]. Unfortunately, the rBWs with the highest overall distortion magnitude for MPRAGE (150 Hz/px and 200 Hz/px for 1.5 T and 3 T scanner, respectively) are used at the current clinical protocol due to aiming for high SNR, which shows that the geometric distortions—when clinically relevant—should be closely controlled in the protocol optimization for these sequences. This also suggests an interconnection between centering and rBW selection: at high SNR regime the distortions may become more strongly dependent on the correct positioning. The effects from mispositioning and rBW to geometric distortions are additive, and the optimal result requires the minimization of both.

As the distortions typically increase with the distance from the isocenter, we summarized our spatially dependent findings by plotting the slopes of the linear fit to the radial distortions against the table position or rBW. This procedure reduced the spatial behavior of the distortions into single values. The slope values and the distortion magnitude distributions were consistent with each other apart from a few exceptions with 3D FLAIR acquisitions (H20-mm-offset MRI-to-CT phantom registration on the 3 T scanner and F20-mm-offset MRI-to-MRI phantom registration on the 1.5 T scanner). However, the distortion magnitudes appear to increase progressively with larger distances from the imaging origin, which limits the applicability of the slope values beyond the presented VOI. The variations present in the slope values imply that the rate-of-change in the distortions, and consequently maximum distortion values, could change unexpectedly with a small change in the imaging settings.

The 3D FLAIR phantom acquisitions suffered from high-intensity variations at the phantom grid edges, indicating that the phantom material or manufacturing process or both were better suited for T1-weighted than FLAIR imaging. The high-intensity artefacts likely originated from intravoxel magnetic-field-homogeneity variations at the boundaries between the signal-producing filling material and the signal-suppressing grid, which induce variable magnetization flip angles and imperfect spoiling of free-induction-decay signals. The appearance of the artefacts could also be affected by the Gibb’s ringing, slice interpolation, partial Fourier or other image reconstruction related effects. We would expect that for instance a template-matching-based control-point-detection algorithm would not endure these artefacts. Previously, we have shown the phantom and nonrigid registration to be a valid method with T1-weighted 3D images [34], and in general, the results showed highly uniform performance between the T1-weighted MPRAGE and the 3D FLAIR acquisitions. An exception to this uniformity was found at rBW = 750 Hz/px, where the relative 3D FLAIR distortions differed from those of the MPRAGE acquisition. We assume that this problem arose from two factors: the high-intensity artefacts were almost aligned between the baseline and the 750-Hz/px-volume, and the overall relative geometric distortions were already small. Thus, the nonrigid registration tried to match the intensity variations. We also detected a rare possibility that a phantom-grid line could jump to a neighboring line in the nonrigid registration if high-intensity artefacts were present, producing excessive distortion magnitudes. No such jumping occurred in the final results, which was visually verified. The image quality of the test-subject acquisitions was sufficient in all the acquisitions, and no problems arose in these registrations. Thus, the test-subject-based method could be more robust for characterizing different kinds of clinical imaging sequences, which encourages further research for using anatomical images in adjunct to phantom images for characterizing and monitoring geometric distortions.

The test subjects were well motivated and co-operative with normal physiological movement, and there were neither planned nor unplanned pauses within dataset acquisitions. Small head movements and table-movement inaccuracies were handled with the rigid registrations, and the resulting translation magnitudes were used for evaluating the inter-acquisition movements. Changes in the measured phantom positions can be attributed to the methods accuracy (< 0.3 mm) and table movement accuracy (< 1.1 mm). The larger values in the test-subject acquisitions underline the importance of the preliminary rigid registration steps.

In this study, the distortion magnitudes were estimated in a limited 80-mm-radius spherical VOI, which approximately corresponds to the size of an adult head and the phantom in use. As the distortions increase progressively within ± 60 mm offset of the imaging origin from the scanner isocenter, the distortions are expected to increase drastically beyond the applied VOI. Furthermore, large distortions could be expected for example in the anterior part of the frontal lobe with notable air-tissue boundaries and magnetic permeability differences, but this area was outside our VOI. In addition, our study includes only two scanners, two clinical sequences, and a single test subject per data set, whereas other anatomical locations may also require registration optimization.

The geometric distortions present in the clinical MRI should be evaluated and minimized when the images are used as a basis for high-accuracy medical operations. The minimization is only possible if there are credible means to evaluate the distortion magnitudes. The presented method can be used to evaluate the change in geometric accuracy between acquisitions of differing imaging parameters, and the presented results offer an estimate of typical error with common imaging setup. As the distortions differ between phantom and clinical imaging, and phantom acquisitions have limitations considering some sequences, a comprehensive image quality assessment should include an investigation of the clinical images alongside the phantom images. The vendor-provided distortion correction had only a limited effect on the distortion evaluation in clinical images. The presented methodology can be extended further to cover additional scanner-sequence combinations.
